# Ultrastructural characterization of primary cilia in pathologically characterized human glioblastoma multiforme (GBM) tumors

**DOI:** 10.1186/1472-6890-14-40

**Published:** 2014-09-12

**Authors:** Joanna J Moser, Marvin J Fritzler, Jerome B Rattner

**Affiliations:** 1 Department of Biochemistry and Molecular Biology, Faculty of Medicine, University of Calgary, Calgary, AB, Canada

**Keywords:** Primary cilia, Ciliogenesis, Cilium-pit, Centriole, Basal body, Distal appendages, Glioblastoma multiforme, EGFR amplification, IDH1/2 mutation, MGMT promoter methylation

## Abstract

**Background:**

Primary cilia are non-motile sensory cytoplasmic organelles that are involved in cell cycle progression. Ultrastructurally, the primary cilium region is complex, with normal ciliogenesis progressing through five distinct morphological stages in human astrocytes. Defects in early stages of ciliogenesis are key features of astrocytoma/glioblastoma cell lines and provided the impetus for the current study which describes the morphology of primary cilia in molecularly characterized human glioblastoma multiforme (GBM) tumors.

**Methods:**

Seven surgically resected human GBM tissue samples were molecularly characterized according to IDH1/2 mutation status, EGFR amplification status and MGMT promoter methylation status and were examined for primary cilia expression and structure using indirect immunofluorescence and electron microscopy.

**Results:**

We report for the first time that primary cilia are disrupted in the early stages of ciliogenesis in human GBM tumors. We confirm that immature primary cilia and basal bodies/centrioles have aberrant ciliogenesis characteristics including absent paired vesicles, misshaped/swollen vesicular hats, abnormal configuration of distal appendages, and discontinuity of centriole microtubular blades. Additionally, the transition zone plate is able to form in the absence of paired vesicles on the distal end of the basal body and when a cilium progresses beyond the early stages of ciliogenesis, it has electron dense material clumped along the transition zone and a darkening of the microtubules at the proximal end of the cilium.

**Conclusions:**

Primary cilia play a role in a variety of human cancers. Previously primary cilia structure was perturbed in cultured cell lines derived from astrocytomas/glioblastomas; however there was always some question as to whether these findings were a cell culture phenomena. In this study we confirm that disruptions in ciliogenesis at early stages do occur in GBM tumors and that these ultrastructural findings bear resemblance to those previously observed in cell cultures. This is the first study to demonstrate that defects in cilia expression and function are a true hallmark of GBM tumors and correlate with their unrestrained growth. A review of the current ultrastructural profiles in the literature provides suggestions as to the best possible candidate protein that underlies defects in the early stages of ciliogenesis within GBM tumors.

## Background

Primary cilia are non-motile sensory cytoplasmic organelles that have been implicated in signal transduction, cell to cell communication, left and right pattern embryonic development, sensation of fluid flow, regulation of calcium levels, mechanosensation, growth factor signaling and cell cycle progression [1,2]. They are present in the central nervous system and depletion of primary cilia in pro-opiomelanocortin hypothalamic neurons have induced hyperphagia [3,4]. Central nervous system primary cilia are key organelles required for Sonic hedgehog signalling (Shh) [5-8] where components Patched, Smoothened, Suppressor of fused and Gli transcription factors concentrate in the primary cilium [9-11]. It is currently thought that an intact primary cilium is required to enable proper Shh pathway function [12]. Subventricular zone astrocytes extend their primary cilium into the cerebral spinal fluid (CSF) suggesting they play a role in sensing ion concentration, pH, osmolarity, and changes in protein or glucose levels [13]. It is possible that astrocyte primary cilia can sense concentrations of neurotransmitters, growth factors, hormones, osmolarity, ions, pH and fluid flow in the extracellular space and relay homeostatic information (or lack thereof) to the centrosome.

Defects in the formation and/or function of primary cilia underlie a variety of human diseases that impact neurological development and are broadly referred to as ciliopathies and include diseases such as Alström, Bardet-Biedl, Joubert, Meckel-Gruber and oral-facial-digital type 1 syndromes. Common neurological phenotypes include obesity, ataxia and mental retardation [14]. The expression and function of primary cilia has become a focus of attention in a number of normal and malignant cells and tissues but have not been characterized in human glioblastoma tissue samples. Given that primary cilia are linked to cell cycle regulation and progression, several studies have suggested that primary cilia may play a role in tumor formation [15,16].

**Figure 1:**
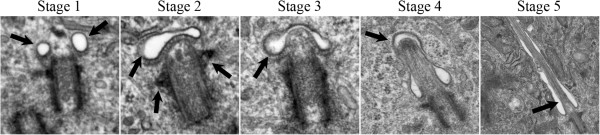
**Primary ciliogenesis progresses through five morphologically distinct stages in human astrocytes.** Key characteristics of each stage are indicated with arrows. Paired lateral vesicles are prominent at the distal end of the basal body in Stage 1. Distal appendages are triangular in appearance and reside at the distal end of the basal body (Stage 2). The paired lateral vesicles fuse to become a vesicular hat and become stretched by the outgrowth of the primary cilium and can be seen progressing through stages 2 through 4. Stage 5 shows a mature primary cilium with a surrounding cilium-pit. *Used with permission from Moser et al. BMC Cancer 2009*, *9*:*448*, *Figure *2*A* © *BioMed Central*.

Previously our group undertook a comparative investigation of primary cilia in cultured primary human astrocytes and compared them to those found in five human astrocytoma/glioblastoma cell lines [17]. We demonstrated that the primary cilium region in cultured astrocyte cells is structurally complex, with ciliogenesis progressing through five distinct stages (Figure 1), and included foci for endocytosis-based signalling [17].

Further, we documented that in each of the five astrocytoma/glioblastoma cell lines studied (U-87 MG, T98G, U-251 MG, U-373 MG, U-138 MG), fully formed primary cilia are either expressed at a very low level, are completely absent or do not proceed through all the stages of ciliogenesis [17]. In addition, we noted several defects in the structure of astrocytoma/glioblastoma centrioles, including abnormal length and appendage architecture, that were not observed in primary human astrocytes [17]. We concluded that aberrant ciliogenesis is common in cells derived from astrocytomas/glioblastomas and that this deficiency likely contributes to the phenotype of these malignant cells. These initial studies in astrocytoma/glioblastoma cell lines indicate that defects in primary cilium ciliogenesis do occur in glioblastoma cells and provided the impetus for this current study which characterizes the morphology of primary cilia and documents ciliogenesis defects in molecularly characterized human glioblastoma multiforme (GBM) tumors. Glioblastomas, although relatively uncommon with an annual incidence rate of 3–4 cases per 100,000 people, have disproportionately high morbidity and mortality rates with median survival pegged at 12–15 months [18,19]. Primary glioblastomas typically occur in patients older than 50 years of age and are characterized by epidermal growth factor receptor (EGFR) amplification and mutations, loss of heterozygosity of chromosome 10q and other abnormalities as reviewed in Wen and Kesari (2008) [20]. One of the most common defects in growth factor signalling involves EGFR [21] and amplification occurs almost exclusively in glioblastomas with 40-50% of patients containing EGFR amplification [20]. Isocitrate dehydrogenase (IDH) mutations are a strong predictor of a more favourable prognosis and a highly selective molecular marker for secondary glioblastomas [22]. Mutations of genes encoding *IDH1* and *IDH2*, as compared to no mutations, are associated with younger age and a better prognosis in adults with gliomas [23]. O^6^-methylguanine-DNA methyltransferase (MGMT) promoter methylation silences the *MGMT* gene, decreasing DNA repair activity and increases the susceptibility of tumor cells to chemotherapeutic agents [20]. Recently it was shown that MGMT promoter methylation was associated with better overall survival in patients with GBM regardless of therapeutic intervention [24]. Given this burden of disease, it is important to determine the degree to which ciliogenesis is compromised in glioblastoma tumors as this information will inform the identity of altered mechanisms which may become targets for the development of future treatments.

Our hypothesis was that primary cilia in human GBM cells would be completely absent or show defects in the early stages of ciliogenesis. Our primary objective was to examine primary cilia expression and structure in human GBM tissue samples at both the light and ultrastructural level.

## Methods

### Ethics statement

Anonymized human brain tumor (GBM) tissue samples and basic clinico-pathologic data were obtained through the Clark Smith Brain Tumour and Tissue Bank at the University of Calgary and Calgary Laboratory Services, Calgary, AB (ethics approved for biobanking and previous patient consent granted at time of banking). Tissue was used according to the policies of the institutional review boards of Calgary Laboratory Services and the Calgary Health Region Ethics Board. Further ethics review and approval for this study (ID# E-23011) was provided by the Conjoint Health Research Ethics Board (University of Calgary, Calgary, AB).

### GBM tissue samples

All samples were part of routine clinical care for diagnostic and treatment purposes and were designated as excess material by the consulting and consenting neuropathologist. Hematoxylin and eosin stained formalin-fixed paraffin-embedded sections were reviewed by a neuropathologist for confirmation of a diagnosis of high-grade glioma (glioblastoma WHO grade IV) as per World Health Organization criteria [18].

### Molecular characterization of GBM tumors

Molecular characterization of IDH and EGFR was performed by the clinical Molecular Diagnostics Laboratory at Calgary Laboratory Services on formalin-fixed paraffin-embedded (FFPE) sections. Briefly, IDH1 and IDH2 mutational analyses were performed using a multiplexed SNaPshot® reaction and detection by capillary electrophoresis [25]. Analysis of EGFR amplification was performed by EGFR colorimetric *in situ* hybridization using standard methods with the EGFR probe #84-1300 (Zymed Laboratories), and scored by a neuropathologist as follows: ‘amplified EGFR’ indicates >10 signals/nucleus in >80% of tumor cells and ‘not amplified’ indicates 2 signals/nucleus in tumor cells.

### Antigen retrieval method (ARM)

Slides containing the FFPE tissue sections were deparaffinised in xylene and passed through a graded ethanol series, rinsed with cold tap water and transferred to a Coplin jar on a hot plate containing a 100°C Tris-EDTA-Tween (w/v) solution (0.121% Tris HCl, 0.0379% EDTA, 0.05% Tween-20, pH adjusted to 9.0). The sections were boiled for 60 minutes and then allowed to reach room temperature while remaining in the same solution. The slides were washed in phosphate buffered saline (PBS) for 10 minutes and processed for IIF.

### Indirect immunofluorescence (IIF)

Formalin-fixed paraffin embedded tissue sections were treated with the above ARM (section 3.4.). Cells were blocked in 10% normal goat serum (NGS; Antibodies Incorporated, Davis, CA) and 2% bovine serum albumin (BSA; Sigma-Aldrich) for 30 minutes at room temperature (RT) and incubated with primary antibodies at appropriate working dilutions overnight at 4°C. Primary cilia were marked by mouse anti-acetylated tubulin at 1:100 dilution (Sigma, St. Louis, MO). After washing with PBS, cells were incubated for 2 hours in a dark chamber with Alexa Fluor (AF) 488 (green) secondary goat fluorochrome-conjugated antibodies at 1:100 dilution (Invitrogen). Slides were washed in several changes of PBS, cell nuclei counterstained with 4’,6-diamidino-2-phenylindole (DAPI), mounted in Vectashield (Vector Laboratories, Burlingame, CA) and examined for IIF using a 100x objective on a Leica DMRE microscope equipped with epifluorescence and an Optronics camera. Appropriate IIF controls with no primary antibody revealed no detectable bleed-through between microscope filter sets.

### Electron microscopy (EM)

Fresh GBM samples were immersed in a fixative containing 3% glutaraldehyde in Millonig’s phosphate buffer and stored at 4°C for 48 hours. Samples were immersed post-fixation in 2% OsO_4_ for 20 minutes and then dehydrated in ethanol and infiltrated with Polybed 812 resin (Polysciences Inc., Warrington, PA). Polymerization was performed at 37°C for 24 hours. Silver-gray sections were cut with an ultramicrotome (Leica) equipped with a diamond knife, stained with uranyl acetate and lead citrate and then examined in a H-700 Hitachi electron microscope. For each sample, 10 grids were examined on standard sections. Approximately 500 cells were examined in each tissue sample.

## Results

We examined both formalin-fixed paraffin embedded (FFPE) and fresh-fixed tissue from seven cases of surgically resected brain tumors diagnosed by neuropathologists as grade IV glioblastoma/GBM using indirect immunofluorescence (IIF) and electron microscopy (EM), respectively.

The GBM tumors were molecularly characterized according to IDH 1/2 mutation status, EGFR amplification status and MGMT promoter status (Table 1). Our results showed that 71% of GBM patients had amplified levels of EGFR, 86% had no IDH1/2 mutations and 50% had methylated MGMT promoters (Table 1).

**Table 1:** Molecularly characterized grade IV glioblastoma GBM tumors

**Patient no.**	**IDH 1/2**	**EGFR amplification**	**MGMT promoter**
1	No mutations detected	Amplified	Not assessed
2	No mutations detected	Amplified	Un-methylated
3	No mutations detected	Amplified	Un-methylated
4	IDH1 exon 4 R132H mutation detected; no mutation in exon 4 of IDH2	Amplified	Methylated
5	No mutations detected	Not amplified	Methylated
6	No mutations detected	Amplified	Methylated
7	No mutations detected	Not amplified	Un-methylated

**Figure 2:**
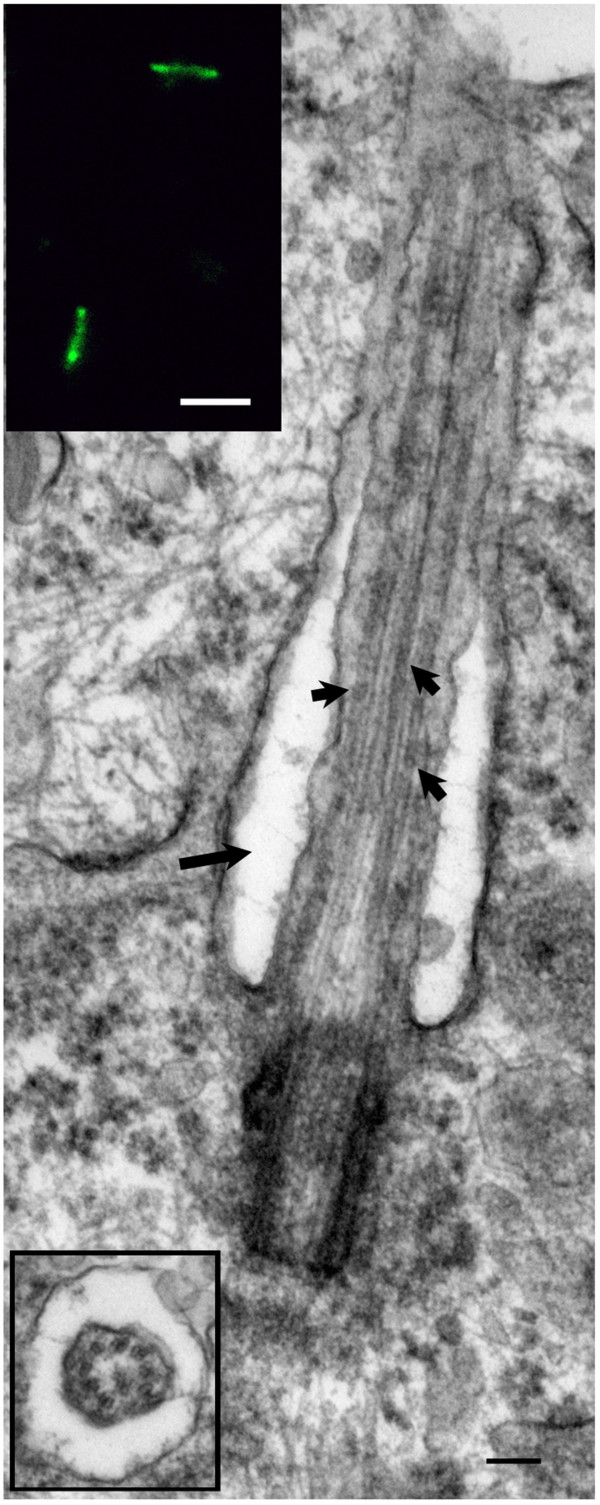
**Patient #1.** GBM cells have an intact primary cilium. Electron micrograph showing a mature primary cilium and basal body with a well-formed cilium-pit (arrow) and endocytotic vesicles (short arrows). Top inset, primary cilia as marked by acetylated tubulin (green) using indirect immunofluorescence analysis. Bottom inset, cross section through the axoneme of another cell. EM scale bar = 100 nm, IIF scale bar = 7 μm.

**Figure 3:**
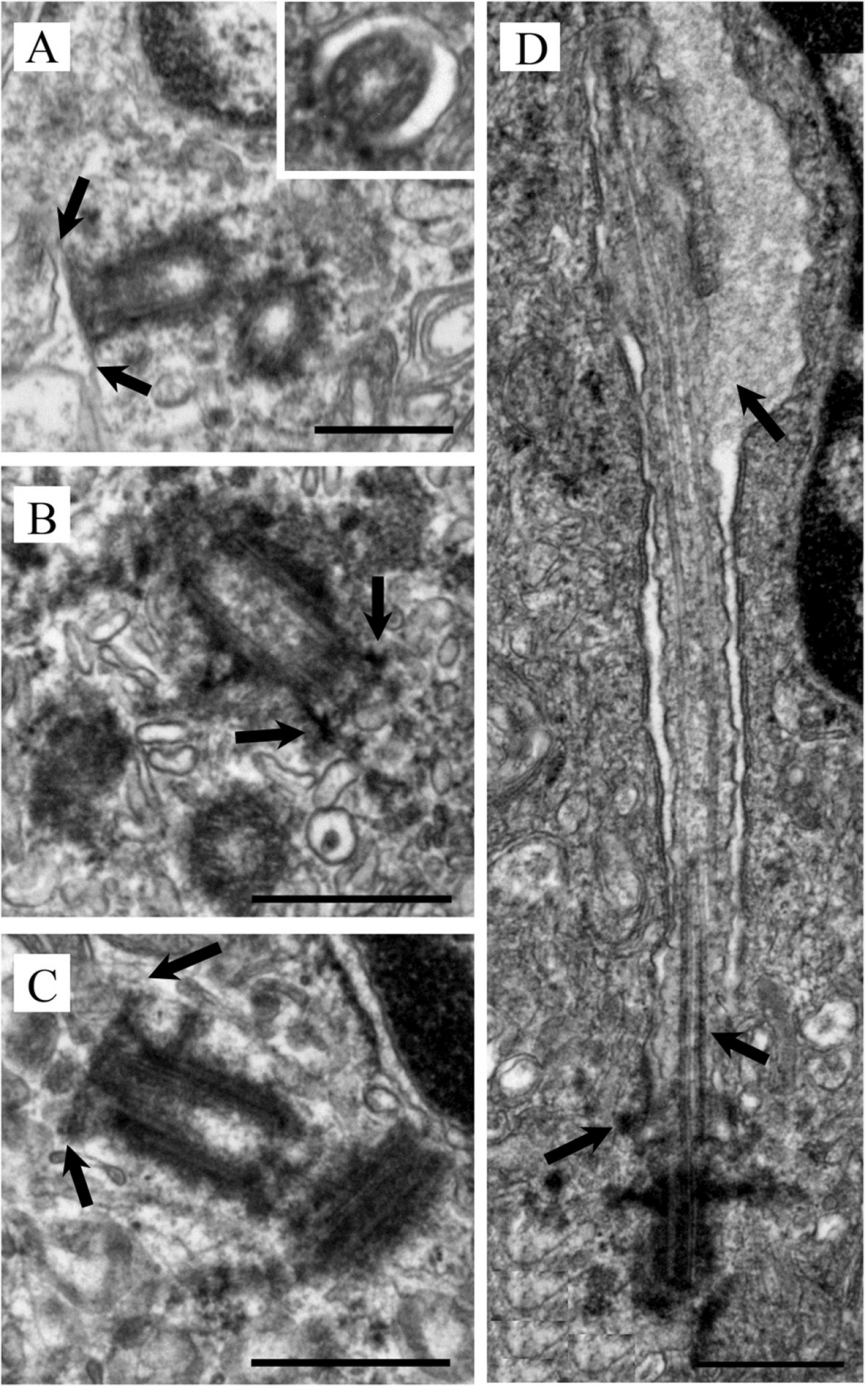
**Patient #2.** GBM cells are halted at stage 1 of ciliogenesis with rare cells progressing to stage 5. **(A)** Basal body and abnormal stage 1 cilium with absent paired lateral vesicles. Inset cross section through the transition zone from another cell. **(B)** Basal body and abnormal stage 1 cilium with vertically outstretched distal appendages and no vesicles. **(C)** Basal body with clear transition zone and abnormal stage 1 cilium with absent vesicles. **(D)** Rare occurrence of a primary cilium at stage 5 with abnormal destruction of the cilium-pit with cytoplasmic extrusion, darkened microtubules at proximal end of cilium and electron dense collection of material at the transition zone. EM scale bars = 500 nm.

**Figure 4:**
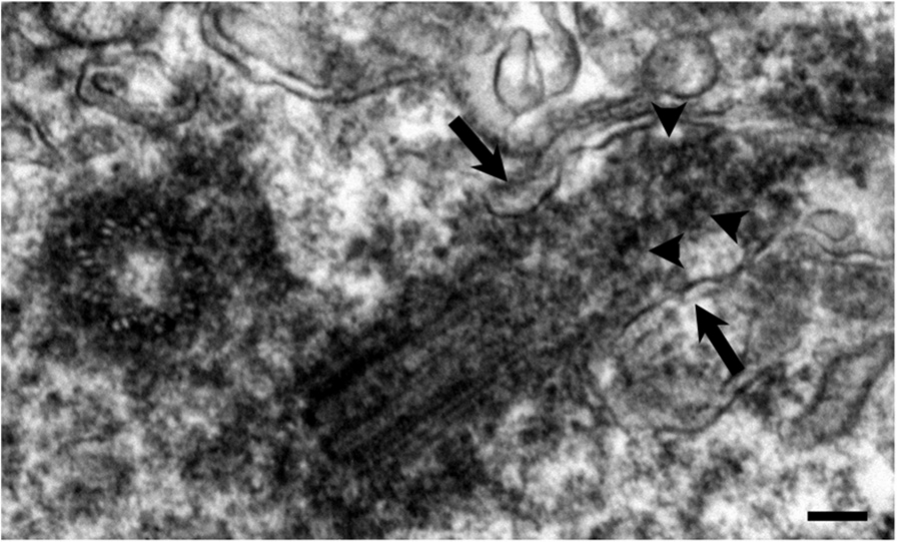
**Patient #3.** GBM cells are halted at stage 3 of ciliogenesis and display electron dense material clustered along the cilium shaft (arrow heads) and an irregular vesicular hat (arrows). EM scale bar = 100 nm.

We examined the biopsy tissue from each of the 7 patients by light and electron microscopy. IIF examination of tissue from patient #1 showed typical primary cilia (Figure 2, top inset). Similarly, ultrastructural examination revealed a normal basal body with a fully formed mature primary cilium, consistent with normal morphology (Figure 2 compared to Figure 1). The cilium-pit was well defined (Figure 2) and the cilium contained well-formed microtubules with normal spacing between doublets (Figure 2, bottom inset). In addition, small vesicles were seen along the cilium microtubules and interfacing with the cilium-pit (Figure 2), which is consistent with previous findings that showed this is a site for endocytosis based signalling [17].The GBM tissue from patient #2 failed to show abundant primary cilia by IIF. Ultrastructural examination revealed cells with basal bodies reminiscent of stage 1 ciliogenesis, however there were no paired vesicles present along the lateral sides of the distal end of the basal body/transition zone as observed in longitudinal and cross-sections (Figure 3A and inset compared to Figure 1). In addition to missing paired vesicles, patient #2 had basal bodies that presented with abnormal, vertically outstretched distal appendages (Figure 3B). Figure 3C shows another example of an abnormal basal body with absent paired vesicles along the lateral sides of the distal end of the basal body/transition zone. Interestingly, the transition zone plate is present without the presence of the vesicles which suggests that the vesicles do not need to be present to allow the transition zone to form, but need to be present to allow ciliogenesis to progress beyond stage 1. In one rare example, a primary cilium which had progressed to stage 5 of ciliogenesis was found (Figure 3D). On close examination, this cilium displayed a disrupted ciliary membrane which was also the site of cytoplasmic extrusions into the surrounding environment (Figure 3D). These abnormal primary cilia also have a dark pericentriolar material (PCM)-like collection of material clumped along the transition zone of the primary cilium with darkening of the cilia microtubules at the proximal end of the cilium shaft (Figure 3D).The tissue from patient #3 revealed an absence of mature primary cilia by IIF and ultrastructural examination showed that 70% of centrosome/basal body profiles were at stage 1 while the remaining 30% of profiles examined displayed stage 2/3 of ciliogenesis (Figure 4). Many of the immature cilia contained electron dense material along the cilium shaft (Figure 4 compared to Figure 1). The stretched vesicular hat that is so prominent in normal cells at stage 2/3 was irregular, thin and misshapen in patient #3 GBM cells (Figure 4). The microtubules of the cilium appear irregular, lack organization and do not display the normal architectural characteristics of the transition zone (Figure 4). There were no cilia in stages 4–5 observed for patient #3.

**Figure 5:**
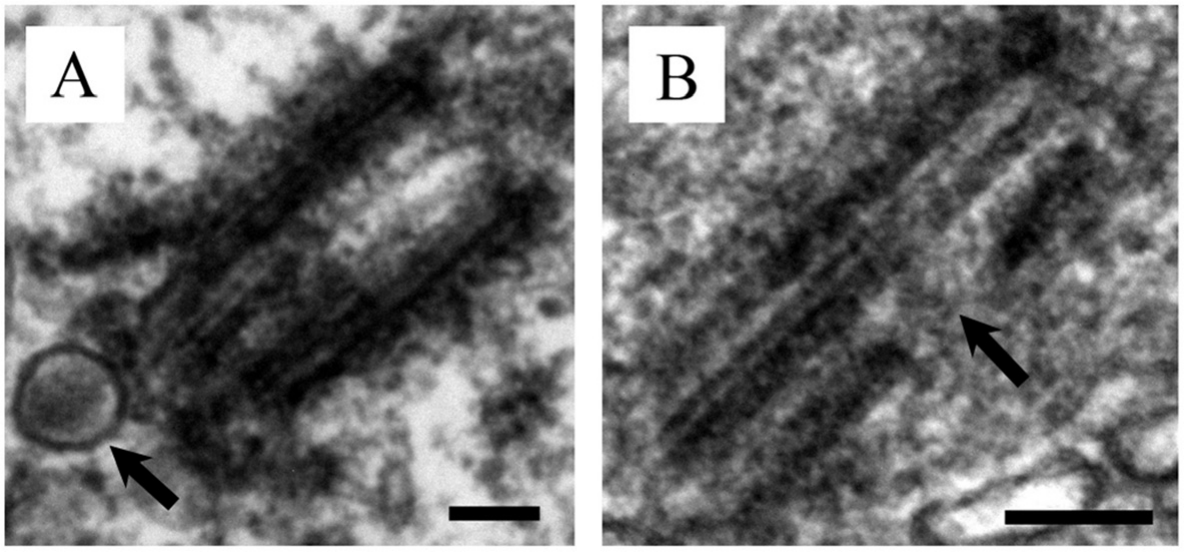
**Patient #4.** GBM cells were characterized by **(A)** primary cilia that were halted at stage 1 of ciliogenesis with a single vesicle present above the transition plate and abnormal distal appendages and **(B)** breakages in the basal body/centriole microtubules. EM scale bars = 100 nm.

**Figure 6:**
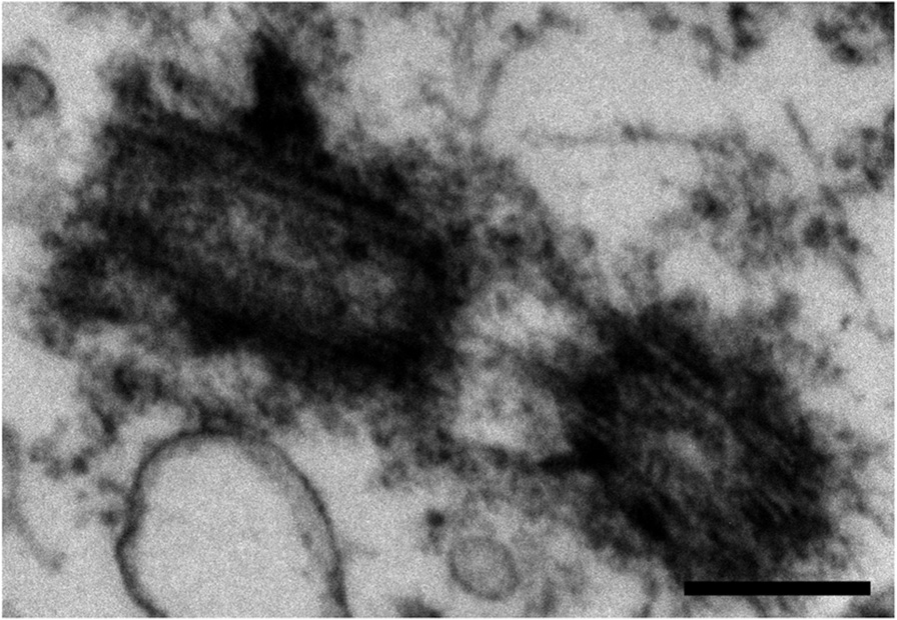
**Patient #5.** GBM cells were characterized by abnormal primary cilia that were halted at or before stage 1 of ciliogenesis with no evidence of paired vesicles or a transition zone plate. EM scale bar = 250 nm.

GBM tissue from patient #4 failed to display mature primary cilium by IIF. Ultrastructurally, patient #4 expressed basal bodies that were similar to stage 1 with a transition zone plate formed along the distal end (Figure 5A compared to Figure 1). It is important to note that in many of these electron microscope centrosome/basal body profiles there was only 1 vesicle present (as opposed to the normal 2 vesicles) at the distal end and the vesicle was positioned directly above the transition zone plate (as opposed to the normal lateral orientation beside the transition zone plate) (Figure 5A). The architecture of the distal appendages along the basal body was also abnormal given their outstretched vertical appearance (Figure 5A) as opposed to the normal horizontal appearance displayed in Figure 1. Figure 5B, illustrates a discontinuity present in one of the centriole microtubule blades, although this centriole was 357 nm in length (Figure 5B) which falls within normal parameters [17].Samples from patient #5 did not show mature primary cilia by IIF. Ultrastructural examination revealed either basal bodies with an immature transition zone or profiles similar to stage 1 (Figure 6 compared to Figure 1). The transition zone was not visible in the electron micrograph centrosome/basal body profiles from this patient and we did not observe any paired vesicles similar to that seen in patient #2 (Figure 6). There also appears to be minimal PCM distributed in the cytoplasmic area surrounding the basal body and centriole (Figure 6).

**Figure 7:**
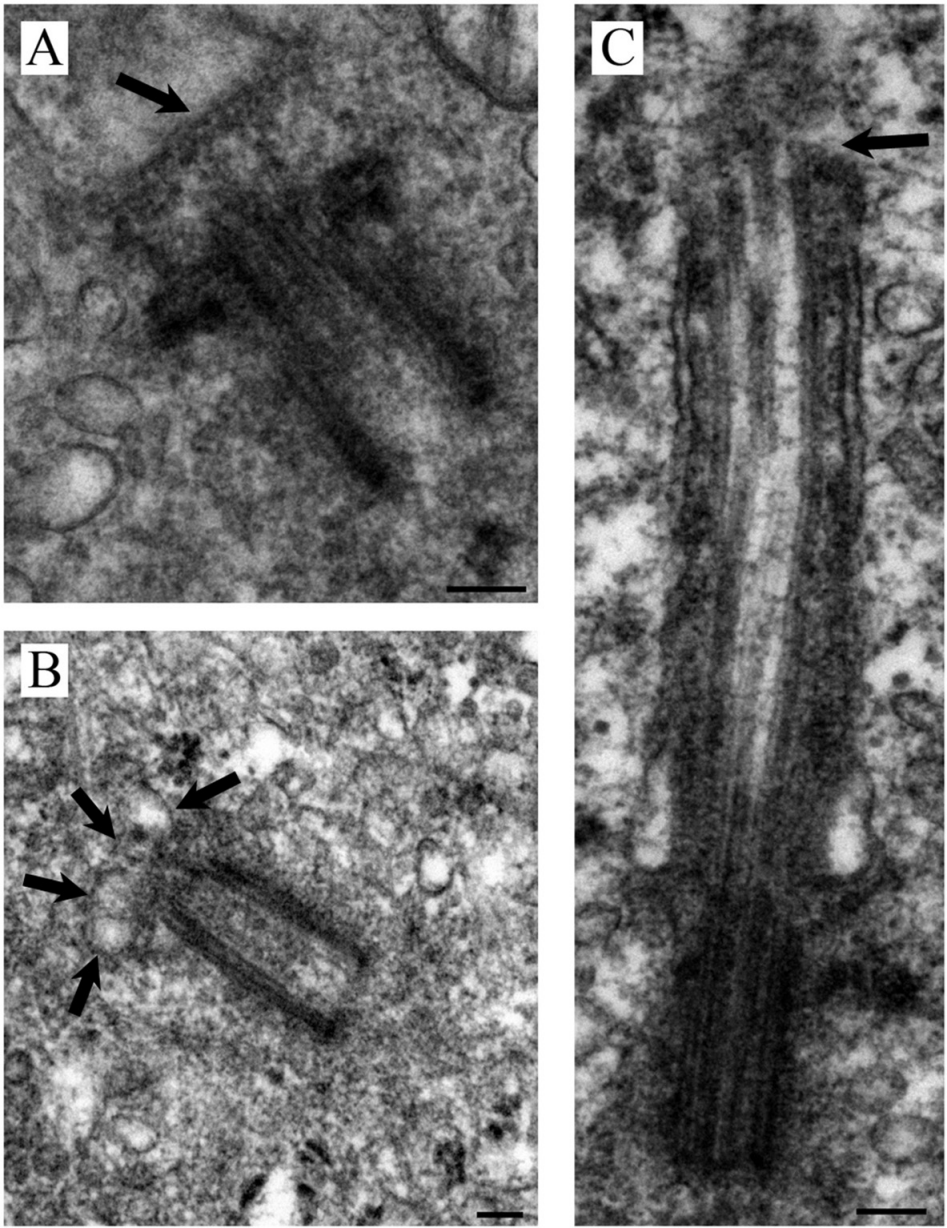
**Patient #6.** GBM cells were characterized by abnormal primary cilia at **(A, B)** stage 1/2 and **(C)** 4/5 of ciliogenesis with either absent lateral vesicles, aberrant supernumerary vesicles along the length of the transition zone plate or abrupt cessation of the cilium shaft. EM scale bars = 100 nm.

There were no observable primary cilia staining by IIF in the GBM tissue from patient #6, although ultrastructural examination showed cilia at multiple stages (Figure 7). We observed profiles at stage 1 with a well-defined plate within the transition zone lacking paired laterally placed vesicles (Figure 7A). We observed many cilia at stage 1 with either multiple irregular abnormally shaped vesicles formed at the distal end of the basal body or 4 distinct vesicles above the plate within the transition zone (Figure 7B). In the latter case, distal appendages were absent. In rare cases, a cilium with a short axoneme was detected (Figure 7C). These cilia appeared to have a truncated cilium-pit so that the distal end of the cilium is continuous with the cytoplasm, a configuration reminiscent of a regressing cilium [26].

**Figure 8:**
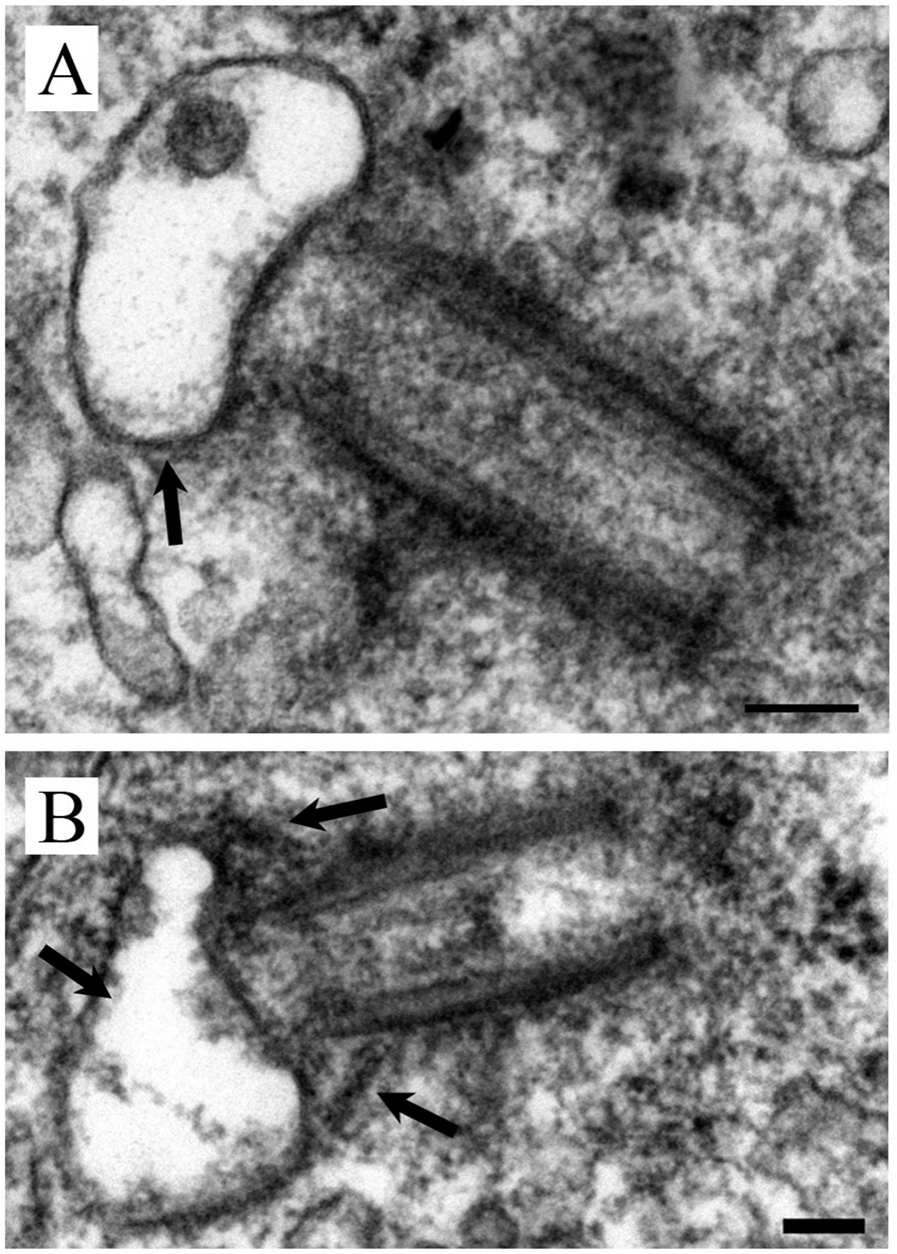
**Patient #7.** GBM cells were characterized by abnormal primary cilia at stage 2 of ciliogenesis with **(A)** swollen vesicular hats **(B)** misshaped vesicular hats and abnormal distal basal body appendages. EM scale bars = 100 nm.

GBM tissue from patient #7 also did not show any primary cilia by IIF staining. Ultrastructurally, primary ciliogenesis occurred in profiles to a maximum of stage 2 (Figure 8 compared to Figure 1). These cells had vesicular hats that were misshaped and swollen (Figure 8A and B) and had outstretched, vertical distal basal body appendages (Figure 8B). These characteristics were similar to those observed in other astrocytoma/glioblastoma cell lines, particularly in U-373 MG and U-138 MG cells (Figure 2B in [17]).

## Discussion

The expression of a primary cilium relies on two main events: 1) activation of ciliogenesis and 2) orderly progression through a series of developmental stages so that a structurally and functionally competent mature cilium is formed [27-29]. Our study illustrates that ciliogenesis was activated in all the GBM samples examined but cilium morphogenesis beyond stage 1 was rare in the majority of tumors. These findings support our previous examination of several astrocytoma/glioblastoma cell lines [17]. Thus, cells from each of these sources (cell line or tumor tissue) express a similar defect or set of defects that targets the earliest stages of ciliogenesis and does not inhibit the cells ability to proliferate.

These findings are compatible with previous studies of melanoma, renal cell carcinoma and pancreatic cancer, which found that primary cilia loss was independent of Ki67 staining (cell proliferation marker) suggesting that cilia loss is not the result of altered cellular proliferation rates but rather may be due to aberrations in another mechanism that is inherent to ciliogenesis [30-32]. Yang and colleagues (2013) recently showed that cell cycle-related kinase (CCRK) and its substrate intestinal cell kinase inhibited ciliogenesis in a glioblastoma cell line [33]. Specifically, they showed that dysregulated high levels of CCRK are present in U-251 MG glioblastoma cells whereby knockdown of CCRK led to the formation of primary cilia indicating that CCRK depletion restored primary ciliogenesis [33]. Furthermore, it was demonstrated that the inhibition of ciliogenesis by over-expression of CCRK in U-251 MG glioblastoma cells promoted cell proliferation capacity [33].

From our ultrastructural studies in astrocytoma/glioblastoma cell lines and GBM tumor tissues it is interesting to note that profiles occasionally displayed centriole/basal bodies with structural abnormalities (i.e. altered length or microtubule integrity). This suggests that it is possible for such structural alterations to be tolerated by the cycling cell, perhaps by being repaired, or that these defects underlie further aberrant cancer cell behaviour.

It is important to note that in the majority of previously published studies, IIF alone was used to evaluate ciliogenesis status. This technique alone does not allow for the precise identification or characterization of the earliest stages of ciliogenesis. Thus, truncated cilia such as that seen in a few patients within our study may be more common that previously indicated. Our ultrastructural data not only reveals a defect in early ciliogenesis but also shows that this defect specifically affects the initial elaboration of the distal surface of the basal body and its ability to associate with Golgi derived vesicles. There have been a number of proteins shown to act at the distal end of the basal body, particularly at the distal appendage region, and they include; Cep170 [34], ninein [8,35], ε-tubulin [36], cenexin/ODF2 [37] (likely cenexin1 [38]) and by association Rab8a [39], centriolin/Cep110 [8,40], Cep164 [41] and Cep123 [42] reviewed in [1]. For example, in neuronal primary cilia, B9-C2 containing proteins have been shown to collect at the base of the primary cilium in the transition zone [43-47] and physically interact and with ciliary protein localization [48] (and reviewed in [49]). One B9-C2 family gene in particular named Stumpy (or *B9d2*) is required for mammalian ciliogenesis where knockout mutants displayed near-complete loss of neuronal primary cilia with remaining cilia displaying dysmorphic stump-like ultrastructures [43].

Of particular interest, a distal appendage protein, Cep 123, has recently been shown to be required for initiation of ciliogenesis by modulation of capping the distal end of the mother centriole with a ciliary vesicle [42]. Sillibourne and colleagues (2013) showed that Cep123 is required for assembly of a primary cilium but not the maintenance of the axoneme in human retinal pigment epithelial (RPE1) cells [42]. Depletion of Cep123 using Cep 123 siRNA perturbed ciliary vesicle formation at the distal end of the basal body which suggests that distal appendage proteins are critical for progression of cilia beyond the early stages of ciliogenesis [42]. These knockdown studies are captured in Figure 6B by Sillibourne *et al*. (2013) [42] and are very similar in appearance to the abnormal early stages of ciliogenesis seen in our GBM tumors. Given this high degree of ultrastructural similarity, Cep123 may be the best candidate to explain the defects we observed in GBM tumors. Although a review of the glioblastoma literature does not highlight Cep123 as being defective in patients with GBM tumors, our study suggests that it is a reasonable target for future expression studies and ultrastructural analysis in GBM tumors.

Limitations of the current study are small sample size and lack of normal brain tissue for comparison. Given the small incidence of malignant gliomas per year, we collected samples over a 5 year period and eliminated those samples that were not grade IV glioblastomas/GBM. To respect the ethics of collection of normal human brain tissue from our patients, we compared the GBM patient results to those previously established in normal human astrocyte cells that were used between passages 3–5 in culture [17]. Although 10 grids were examined for each patient sample, there were noticeable differences between tumor samples in terms of cellularity and patient heterogeneity. It is important to emphasize that this is a complex mixture of cells and extracellular matrixof GBM brain tissue and that some cell types are ciliated whereas others are not ciliated. Because of the tissue complexity and heterogeneity, it is impossible to identify all the cells which have the potential to undergo ciliogenesis. When we do see profiles, we can quantitate the number of cells undergoing abnormal ciliogenesis (including profiles at stages 3/4/5) which is summarized as follows. Patient #1: 20 cells with profiles, 2 cells with normal cilia, 0 cells with abnormal cilia. Patient #2: 9 cells with profiles, 0 cells with normal cilia, 2 cells with abnormal cilia. Patient #3: 20 cells with profiles, 0 cells with normal cilia, 2 cells with abnormal cilia. Patient #4: 22 cells with profiles, 0 cells with normal cilia, 0 cells with abnormal cilia. Patient #5: 20 cells with profiles, 0 cells with normal cilia, 0 cells with abnormal cilia. Patient #6: 30 cells with profiles, 0 cells with normal cilia, 3 cells with abnormal cilia. Patient #7: 15 cells with profiles, 0 cells with normal cilia, 0 cells with abnormal cilia. Taken together, only one patient expressed morphologically normal cilia. As a whole, we did see the same types of abnormalities in the early stages of ciliogenesis amongst tumor samples which suggests that early ciliogenesis defects are a generalized problem in GBM tumors. In summary, we can say that the large majority of grade 4 glioblastoma/astrocytomas (i.e. GBM tumors) are likely to express abnormal immature primary cilia suggesting that this defect may be a hallmark of GBMs. We have not detected a clear correlation between abnormal ciliogenesis and the 3 main molecular characterizations examined in these patient samples. It must be kept in mind that our patient sample size may not be sufficient to reveal correlations with molecular markers and this might require a larger study.

In summary, we found that ciliogenesis is activated in GBM tumors but the normal development of a mature cilium is perturbed at early stages of ciliogenesis. The aberrant ultrastructural profiles observed in our survey of GBM tumors and a review of the current ultrastructural profiles present in the literature suggest the possibility that at present the best possible candidate protein underlying defects in the early stages of ciliogenesis within GBM tumors might involve Cep123.

## Conclusions

The major finding of this report is that primary ciliogenesis is disrupted at an early stage in the majority of human GBM tumors. This finding is important for several reasons. First, these results confirm astrocytoma/glioblastoma cell culture data. Second, it indicates that defects in ciliogenesis are a hallmark of GBM tumor pathology and provides impetus for further study of the relationship between primary cilium defects and other brain tumors such as astrocytoma, oligodendroglioma and medulloblastoma. Third, it provides further evidence that the early stages of ciliogenesis are a critical time in the process of ciliogenesis, thus narrowing the number of target proteins that may underlie these defects. Fourth, it catalogues and describes the key basal body/cilium-related ultrastructural abnormalities that are common between GBM tumors. The ultrastructural description of this study informs which proteins are involved in early ciliogenesis defects and identifies candidates that are currently in the literature. In future, it will be important to elucidate which specific proteins are involved in this critical time period and whether alterations in their expression can restore ciliogenesis and thus restore cell cycle control.

## Abbreviations

ARM: Antigen retrieval method; CCRK: Cell cycle-related kinase; CSF: Cerebral spinal fluid; DAPI: 4’,6-diamidino-2-phenylindole; EM: Electron microscopy; EGFR: Epidermal growth factor receptor; FFPE: Formalin-fixed paraffin embedded; GBM: Glioblastoma multiforme; IIF: Indirect immunofluorescence; IDH: Isocitrate dehydrogenase; MGMT: O^6^-methylguanine-DNA methyltransferase; PBS: Phosphate buffered saline; PCM: Pericentriolar material; RPE1: Retinal pigment epithelial; Shh: Sonic hedgehog signalling; WHO: World Health Organization.

## Competing interests

The authors declare that they have no competing interests.

## Authors’ contributions

JJM and JBR obtained the molecular characterization data, carried out the electron microscopy studies, performed indirect immunofluorescence experiments and wrote the first draft of the manuscript. JJM, MJF and JBR conceived of the study design, participated in obtaining ethics approval, interpretation of the data, read, edited and approved the final manuscript. All authors read and approved the final manuscript.

## Pre-publication history

The pre-publication history for this paper can be accessed here:

http://www.biomedcentral.com/1472-6890/14/40/prepub
